# General Practitioners’ vitamin K antagonist monitoring is associated with better blood pressure control in patients with hypertension – a cross-sectional database study

**DOI:** 10.1186/s12872-015-0053-x

**Published:** 2015-06-10

**Authors:** Sven Streit, Vladimir Kaplan, André Busato, Sima Djalali, Oliver Senn, Damian N. Meli, and the FIRE study group

**Affiliations:** Institute of Primary Health Care BIHAM, University of Bern, Gesellschaftsstrasse 49, 3012 Bern, Switzerland; District Hospital Freiamt Muri, Muri, Switzerland; Institute of General Practice, University of Zurich, University Hospital Zurich, Zurich, Switzerland

**Keywords:** Anticoagulant agents, Blood pressure control, Primary care

## Abstract

**Background:**

Patients requiring anticoagulation suffer from comorbidities such as hypertension. On the occasion of INR monitoring, general practitioners (GPs) have the opportunity to control for blood pressure (BP). We aimed to evaluate the impact of Vitamin-K Antagonist (VKA) monitoring by GPs on BP control in patients with hypertension.

**Methods:**

We cross-sectionally analyzed the database of the Swiss Family Medicine ICPC Research using Electronic Medical Records (FIRE) of 60 general practices in a primary care setting in Switzerland. This database includes 113,335 patients who visited their GP between 2009 and 2013. We identified patients with hypertension based on antihypertensive medication prescribed for ≥6 months. We compared patients with VKA for ≥3 months and patients without such treatment regarding BP control. We adjusted for age, sex, observation period, number of consultations and comorbidity.

**Results:**

We identified 4,412 patients with hypertension and blood pressure recordings in the FIRE database. Among these, 569 (12.9 %) were on Phenprocoumon (VKA) and 3,843 (87.1 %) had no anticoagulation. Mean systolic and diastolic BP was significantly lower in the VKA group (130.6 ± 14.9 vs 139.8 ± 15.8 and 76.6 ± 7.9 vs 81.3 ± 9.3 mm Hg) (*p* < 0.001 for both). The difference remained after adjusting for possible confounders. Systolic and diastolic BP were significantly lower in the VKA group, reaching a mean difference of −8.4 mm Hg (95 % CI −9.8 to −7.0 mm Hg) and −1.5 mm Hg (95 % CI −2.3 to −0.7 mm Hg), respectively (*p* < 0.001 for both).

**Conclusions:**

In a large sample of hypertensive patients in Switzerland, VKA treatment was independently associated with better systolic and diastolic BP control. The observed effect could be due to better compliance with antihypertensive medication in patients treated with VKA. Therefore, we conclude to be aware of this possible benefit especially in patients with lower expected compliance and with multimorbidity.

## Background

In times of novel oral anticoagulants (NOACs) coagulation monitoring (International Normalized Ratio testing, INR) is no longer needed. In several studies, NOACs have been shown not to be inferior to VKAs in stroke prevention in patients with atrial fibrillation [1] and in the treatment of venous thromboembolism [2].

However, there are several reasons why General Practitioners (GPs) may hesitate to switch patients from VKA to NOAC if INR is in therapeutic range: the higher price of NOACs, the greater risk of gastrointestinal bleeding [3], being familiar with perioperative (bridging)-management, and lastly no validated strategies for bleeding complications.

In addition, patients requiring anticoagulation often suffer from significant co-morbidities such as hypertension, cardiovascular disease (CVD), diabetes or renal failure [4]. In many countries, the INR value is monitored by GPs [5]. On the occasion of INR monitoring, GPs have the opportunity to provide additional care to patients, such as adjusting the dosage of antihypertensive drugs, giving smoking cessation advice or treating high cholesterol. Therefore, it could be assumed, that patients under VKA treatment and consequent INR monitoring receive more comprehensive care of their comorbidities than patients without VKA treatment due to a higher consultation rate. A similar, but possibly confounding effect has been shown in a large Danish cohort of hypertensive patients, where patients with co-morbidities, particularly patients with heart failure and CVD had a significantly better blood pressure (BP) control [6]. This may indicate that the quality of care increases with the number of conditions causing a higher consultation rate and thus closer care management.

In our study, we aimed to evaluate the specific impact of INR monitoring on BP control in primary care patients with hypertension. Taking into consideration that other chronic conditions needing periodical monitoring could alter observed effects, we also investigated if patients with comorbid diabetes would differ regarding BP control. We focused on diabetes for two reasons. First, it is a chronic disease commonly managed in primary care with internationally well accepted guidelines uniformly suggesting a regular recall of patients for the monitoring of blood sugar target values. Second, it is undisputed that diabetes itself is an indication for BP control. If INR monitoring had a specific impact on BP control, it could be assumed that the effect would also be observed in patients with comorbid diabetes.

## Methods

Primary care patients were identified from the Family medicine ICPC-Research using Electronic medical records (FIRE) project database. The FIRE project is an ongoing research project at the Institute of General Practice of the University of Zurich, Switzerland. Established in 2009, it provides the first and largest standardized collection of structured medical routine data from Swiss primary care. Details about the database structure are reported elsewhere [7]. In brief, the database covers reasons for encounter according to the ICPC-2 classification (International Classification of Primary Care 2) [8], patient demographics, vital signs, laboratory data and both type and dosage of prescribed medication according to Anatomical Therapeutic Chemical/Defined Daily Dose Classification (ATC/DDD) coding established by the WHO [9]. General practitioners participating in the FIRE project extract these data in an anonymized way from their electronic medical records and pool them centrally in the FIRE database aggregated by individual consultation dates.

In our study, we identified patients being treated for hypertension based on prescribed medication. Cases with ATC coding specifying antihypertensive medication were detected following the concept of pharmaceutical cost groups (PCG) [10]. We included all patients with respective medication for ≥6 months, at least two consultations within one year from May 2009 to February 2013 and recorded BP measurements. Subsequently, we selected patients with VKA treatment (ATC coding) for ≥3 months and compared them to patients without such treatment regarding BP control. In Switzerland, INR monitoring is mainly done by GPs using a point-of-care device in order to review the prescription and adjust dosing of VKA during the same consultation. We controlled for potential confounders by performing a linear regression analysis adjusting for age, sex, observation period, number of consultations, chronic conditions, coronary heart disease, heart failure, atherosclerosis, obesity, and diabetes. Chronic conditions were defined by applying a set of specific ICPC-2 codes first described by O’Halloran *et al.* [11] and validated for the FIRE database [12]. Moreover, we identified patients with diabetes type two by prescribed medication (PCG).

Data were analysed using STATA release 13.1 StataCorp, College Station, TX.

According to current Swiss law on human research (Humanforschungsgesetz, HFG) retrospective cross-sectional analysis of anonymized medical routine data requires no approval by the regional ethics committee Zürich [13]. Patient records/informations were anonymized and de-identified prior to analysis.

## Results

**Fig. 1:**
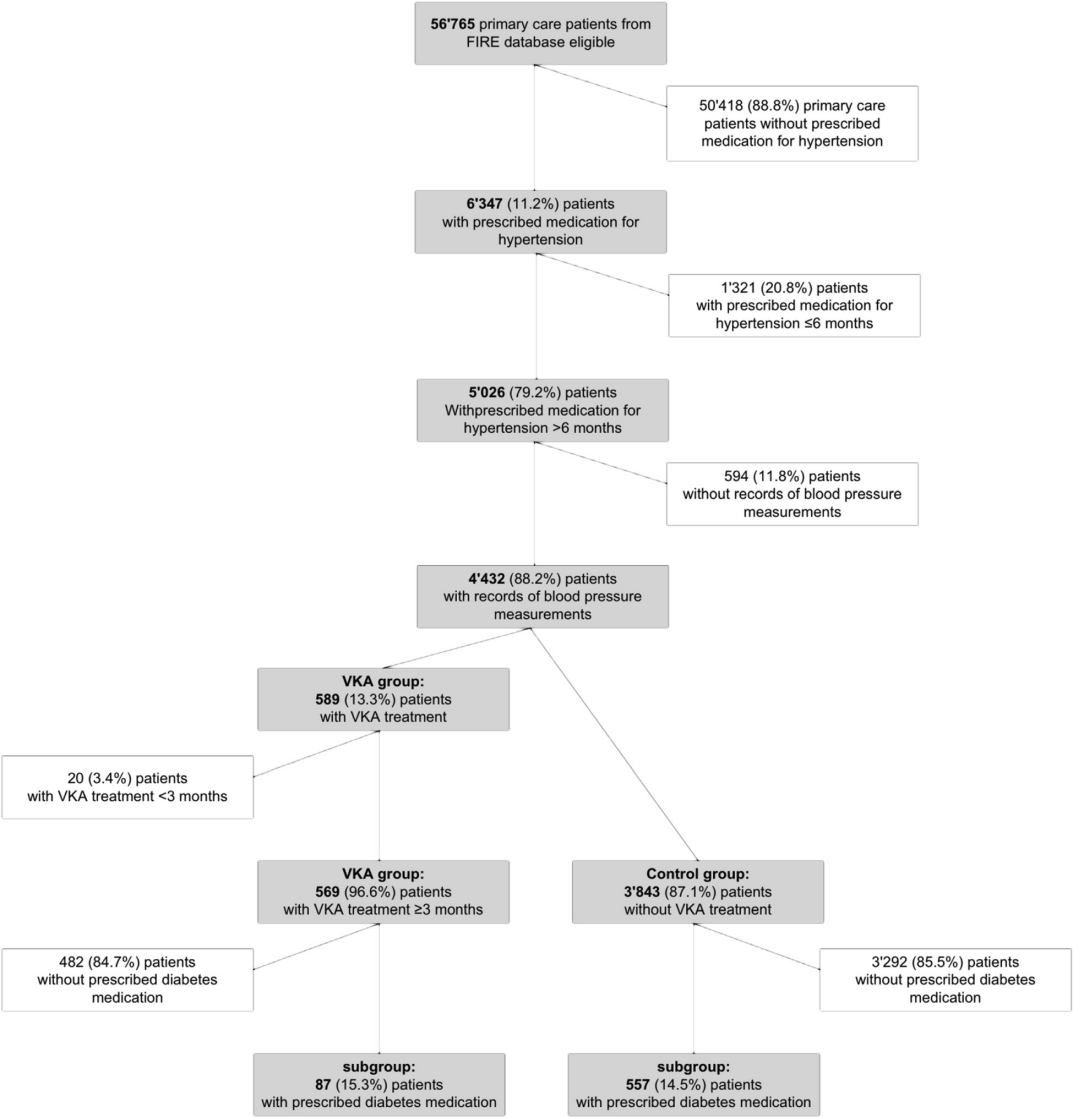
Patient flow chart

Consultation data of 56,765 adult primary care patients with at least two consultations within one year between May 2009 and February 2013 were eligible (Flowchart in Fig. 1). 6,347 of these patients (11.2 %) had a diagnosis of hypertension according to their list of medication. 5,026 (79.2 %) had prescribed medication for hypertension ≥6 months. Out of the 5,026 patients, 4,432 (88.2 %) had records of BP measurements and included in our study. Among these 4,432, 569 (12.9 %) where treated with Phenprocoumon ≥3 months and were included in the VKA group; 3,843 (87.1 %) patients had no anticoagulant treatment and were used as controls.

Table 1 depicts the baseline characteristics of patients in the VKA and control groups. The two groups differed significantly in age, sex, number of consultations per year and number and type of chronic conditions. Patients on VKA were approximately nine years older, more likely to be female, had more chronic comorbidities and visited their GP almost twice as often as controls.

**Table 1:** Baseline characteristics and blood pressure (BP) of 4,412 patients with hypertension with and without VKA Treatment

	VKA group n = 569	Control group n = 3,843	*p*-value**
Age, years (SD)	76.7 (10.0)	67.8 (13.8)	<0.01
Men, %	47.6	52.4	0.044
Consultations/year, n (SD)	10.9 (7.2)	6.6 (5.5)	<0.01
Chronic conditions, n (SD)	3.8 (2.5)	3.1 (2.3)	<0.01
Coronary heart disease, n (%)	45 (7.9)	224 (5.8)	0.053
Heart failure, n (%)	28 (4.9)	50 (1.3)	<0.01
Atherosclerosis, n (%)	28 (4.9)	80 (2.1)	<0.01
Obesity, n (%)	20 (3.5)	235 (6.1)	0.01
Diabetes, n (%)	87 (15.3)	557 (14.5)	0.62
Mean systolic BP, mm Hg (SD)	130.6 (14.9)	139.8 (15.8)	<0.01
Mean diastolic BP, mm Hg (SD)	76.6 (7.9)	81.3 (9.3)	<0.01
Patients with controlled* BP, %	74.9	49.4	<0.01

*defined as blood pressure <140/90 mmHg

**p-value: Results of univariate comparisons between groups based on unpaired *t*-test or Chi-square test as appropriate

Regarding BP control, both mean systolic and diastolic blood pressure were significantly lower by 9.2 mm Hg (systolic) and 4.7 mm Hg (diastolic) in the VKA group (*p* < 0.01 for both) (Table 1). Additionally, the proportion of patients with controlled BP within target range, defined as <140/90 mm Hg, was significantly higher in the VKA group (74.9 % vs. 49.5 %, *p* < 0.01.).

Table 2 provides the mean differences of systolic and diastolic BP between groups after adjustment for age, sex, observation period, number of consultations, number of chronic conditions, coronary heart disease, heart failure, atherosclerosis, obesity, and diabetes. Again, both systolic and diastolic BP were significantly lower in the VKA group, and patients in the VKA group were more likely to meet the BP target range of <140/90 mm Hg, odds ratio 2.7 (95 % CI 2.2 – 3.4).

**Table 2:** Adjusted difference in blood pressure of patients with hypertension with and without VKA Treatment

Patients included (n = 4,412)	VKA group vs. control group	
	Adjusted^a^ mean difference (95 % CI)	*p*-value
Systolic BP (mm Hg)	−8.4 (−9.8 − −7.0)	<0.01
Diastolic BP (mm Hg)	−1.5 (−2.3 − −0.7)	<0.01
	Adjusted* Odds Ratio (95 % CI)	*p*-value
Controlled BP (<140/90 mm Hg)	2.7 (2.2 – 3.4)	<0.001

^a^adjusted for age, sex, observation period, number of consultations and number of chronic conditions, coronary heart disease, heart failure, atherosclerosis, obesity, diabetes

Differences were also observed between the subgroups of patients with comorbid diabetes (n = 644) (Table 3). Mean systolic BP was significantly lower in the VKA group (−7.2 mm Hg, *p* < 0.001); mean diastolic BP was not significantly lower (−0.4 mm Hg, *p* < 0.7). Odds ratio for controlled BP <140/90 mm Hg was 1.7 (95 % CI 1.0 – 2.8, *p* = 0.044).

**Table 3:** Adjusted difference in blood pressure of patients with hypertension and comorbid diabetes with and without VKA Treatment

Subgroup of patients with prescribed diabetes medication (n = 644)	VKA group vs. control group	
	Adjusted^a^ mean difference (95 % CI)	*p*-value
Systolic BP (mm Hg)	−7.2 (−10.9 − −3.6)	<0.001
Diastolic BP (mm Hg)	−0.4 (−2.3 − 1.5)	0.68
	Adjusted^a^ Odds Ratio (95 % CI)	*p*-value
Controlled BP (<140/90 mm Hg)	1.7 (1.01 – 2.8)	0.044

^a^adjusted for age, sex, observation period, number of consultations, number of chronic conditions, coronary heart disease, heart failure, atherosclerosis, obesity, diabetes

## Discussion

The aim of the study was to evaluate the effect of INR monitoring on BP control in primary care patients with hypertension. The results suggest that the hypothesized effect exists and that it is clinically relevant. After adjustment for confounders, both systolic and diastolic BP were significantly lower in the VKA group by about 9 mm Hg and 2 mm Hg, respectively.

These findings lead to the question, ‘Which factor associated with INR monitoring could cause the effect?’ One explanation is that INR monitoring requires regular consultations with the GP, where INR values are available right within the same consultation due to a point-of-care device. Indeed, comparison showed that patients under VKA treatment had significantly more consultations per year with their GP than controls. However, in the multivariate analysis, we controlled for the number of consultations and the effect sustained. Similarly, we controlled for the number of chronic conditions, because one could argue that patients under VKA treatment are more like to suffer from multiple chronic conditions which require regular monitoring and increase GPs’ awareness for BP control. A very common chronic disease that could unfold such a confounding effect is diabetes. In fact, the prevalence of patients with diabetes was approximately 15 % in our study population and therefore in the expected range considering age and morbidity [14]. Moreover, previous studies have shown that the majority of patients with diabetes in Swiss primary care undergo regular BP measurement [15]. Apparently, GPs are aware of the necessity of BP control in patients with diabetes. So, we analyzed patients with comorbid diabetes separately. Again, a lower BP under VKA treatment was observed.

Another explanation is that VKA agents have an inherent BP lowering effect. For instance, animal experiments revealed that specific coumarin agents or compounds have a dose-dependent, BP lowering effect [16, 17]. However, Phenprocoumon is not among these. Moreover, if Phenprocoumon had a physical effect on BP, normotensive patients under VKA treatment would regularly suffer from hypotension. To conclude, we consider this explanation as rather implausible.

Another explanation for our findings is that INR monitoring promotes patients’ adherence to medical therapy and subsequently enhances the intake of antihypertensive medication more than other monitoring measures. If so, it would be interesting to investigate whether the effect could also be observed in patients who routinely receive INR measurement by different healthcare providers or who perform self-monitoring at home.

Our study has several limitations. Due to the design of our study, we can only speculate about causality between VKA treatment and better blood pressure control. We also did not measure compliance to VKA treatment directly because our dataset did not contain INR-values or time in therapeutic range. However, also after adjusting for several possible confounding factors, we still observed a strong association between VKA and lower blood pressure. However, there remains the possibility of residual confounding due to missing data on e.g. BMI and limitations due to undercoding for reasons for anticoagulation.

A major strength of our study is that it is based on the substantial FIRE database and consequently a large sample size. As in February 2013, the database contained records from 56,756 patients and led to the inclusion of 4,412 patients in this study. Moreover, our inclusion criteria were rather strict, defining hypertension as antihypertensive medication intake for at least six months. As newly induced treatments can cause great variations in blood pressure control, we ensured that patients at the beginning of antihypertensive treatment were excluded. Patients who had received VKA treatment for less than three months were also excluded, in order to avoid the inclusion of patients under short-term anticoagulation therapy after thrombosis. Therefore, data can be considered as representative.

A significant association between VKA treatment and BP control could be shown. Although, our study method is limited to a cross-sectional design and not qualified to prove causality, our findings are of special relevance for daily practice in primary care. In the light of an increasingly older and multimorbid population, new models of care are required that are tailored to the needs of chronically ill patients. Indeed, health services research has yielded several promising concepts, e.g. the Chronic Care Model or the Patient-Centered Medical Home Model [18, 19]. However, the implementation of these concepts into primary care routine of European countries is challenging and still in the early stage of its development [20]. Meanwhile, regularly recurring processes of care such as INR monitoring in GP practices fill in the gap and constitute an important opportunity for GPs to structure care provision, evolve a stable physician-patient-relationship and provide comprehensive medical care. Our findings show the importance of this «gap filling» and highlight that VKA should not be simply replaced by NOACs without a thorough implementation of new care models.

Further research is needed, in order to clarify which aspect of INR monitoring is causative for the observed BP lowering effect and how it could be systematically exploited, particularly in multimorbid patients.

## Conclusions

In a large sample of hypertensive patients in Switzerland, Vitamin-K antagonist treatment was associated with better systolic and diastolic BP control even after adjusting for several covariates such as comorbidities and number of consultations. The observed effect could be due to better compliance with antihypertensive medication in patients treated with VKA. Therefore, we conclude to be aware of this possible benefit especially in patients with lower expected compliance and with multimorbidity.
